# Studying Dynamic Myofiber Aggregate Reorientation in Dilated Cardiomyopathy Using In Vivo Magnetic Resonance Diffusion Tensor Imaging

**DOI:** 10.1161/CIRCIMAGING.116.005018

**Published:** 2016-10-18

**Authors:** Constantin von Deuster, Eva Sammut, Liya Asner, David Nordsletten, Pablo Lamata, Christian T. Stoeck, Sebastian Kozerke, Reza Razavi

**Affiliations:** From the Department for Biomedical Engineering, Division of Imaging Sciences and Biomedical Engineering, King’s College London, United Kingdom (C.v.D., E.S., L.A., D.N., P.L., C.T.S, S.K., R.R.); and Department of Information Technology and Electrical Engineering, Institute for Biomedical Engineering, University and ETH Zurich, Switzerland (C.v.D., C.T.S., S.K.).

**Keywords:** diffusion tensor imaging, dilated cardiomyopathy, magnetic resonance imaging, myocardium, myofiber architecture

## Abstract

**Background—:**

The objective of this study is to assess the dynamic alterations of myocardial microstructure and strain between diastole and systole in patients with dilated cardiomyopathy relative to healthy controls using the magnetic resonance diffusion tensor imaging, myocardial tagging, and biomechanical modeling.

**Methods and Results—:**

Dual heart-phase diffusion tensor imaging was successfully performed in 9 patients and 9 controls. Tagging data were acquired for the diffusion tensor strain correction and cardiac motion analysis. Mean diffusivity, fractional anisotropy, and myocyte aggregate orientations were compared between both cohorts. Cardiac function was assessed by left ventricular ejection fraction, torsion, and strain. Computational modeling was used to study the impact of cardiac shape on fiber reorientation and how fiber orientations affect strain. In patients with dilated cardiomyopathy, a more longitudinal orientation of diastolic myofiber aggregates was measured compared with controls. Although a significant steepening of helix angles (HAs) during contraction was found in the controls, consistent change in HAs during contraction was absent in patients. Left ventricular ejection fraction, cardiac torsion, and strain were significantly lower in the patients compared with controls. Computational modeling revealed that the dilated heart results in reduced HA changes compared with a normal heart. Reduced torsion was found to be exacerbated by steeper HAs.

**Conclusions—:**

Diffusion tensor imaging revealed reduced reorientation of myofiber aggregates during cardiac contraction in patients with dilated cardiomyopathy relative to controls. Left ventricular remodeling seems to be an important factor in the changes to myocyte orientation. Steeper HAs are coupled with a worsening in strain and torsion. Overall, the findings provide new insights into the structural alterations in patients with dilated cardiomyopathy.

## Abstract 2

Supplemental Digital Content is available in the text.

Dilated cardiomyopathy (DCM) is a major cause of heart failure, morbidity, and mortality. It is a multifactorial disease encompassing hereditary and acquired forms.^1^ Although heterogeneous in cause, histologically most cardiac findings are nonspecific, with hypertrophy and elongation of myocytes, reduced density of myofibrils, cellular necrosis, and fibrosis.^2^

**See Editorial by Nguyen et al**

**See Clinical Perspective**

The clinical course is variable, however progressive and largely irreversible. The disease is characterized by advancing ventricular chamber enlargement and systolic dysfunction with an increased risk of sudden cardiac death.^3^ Over time, the heart becomes unable to compensate for the loss of contractile force, and clinical manifestations become apparent.^4^ Pathophysiologically, there is myocyte dysfunction and disarray. In addition, activation of neurohormonal pathways exacerbates cardiac hemodynamic anomalies, potentially leading to adverse cardiac remodeling or end organ damage.^5^ Despite developments in understanding and treatment approaches, the disease is not yet fully characterized, and prognosis remains poor.

In recent years, the field of cardiac magnetic resonance (MR) diffusion tensor imaging (DTI) has gained significant momentum. Quantitative information on the orientation of myocardial fiber aggregates from ex vivo DTI has been shown to correlate well with the histological observations.^6,7^ The integrity, mobility, and arrangement of the myocytes contribute significantly to efficient ventricular function,^8^ and cardiac DTI has shown potential to gain novel insights into various cardiac conditions.^9–13^ Using the diffusion-sensitizing MR sequences, the displacement probability of diffusing water molecules within the tissue of interest can be measured, and the arrangement of myocyte aggregates can be inferred using the diffusion tensor calculus. Scalar metrics, such as mean diffusivity (MD) and fractional anisotropy (FA), allow characterization of structural integrity.^11,12,14^

Cardiac DTI has been performed primarily ex vivo.^11,12,15,16^ In preclinical DCM models, alterations of the transmural helix angle (HA) slopes^17^ with increased diffusivity and decreased diffusion anisotropy have been described.^18^ With advances in MR imaging methodology, in vivo cardiac DTI has been shown to be feasible and robust in animal and human studies.^14,19–23^ Moreover, the implementation of dual heart-phase cardiac DTI now also permits insights into dynamic changes of myocardial fiber aggregates during the cardiac cycle confirming and complementing ex vivo studies in hearts fixated in diastolic or systolic states.^7,24^ In vivo dual heart-phase DTI has been demonstrated both in healthy volunteers and in patients with hypertrophic cardiomyopathy.^10,21^

The objective of this work was to study the relative dynamic alterations of myocardial microstructure in patients with DCM and healthy controls using dual heart-phase cardiac DTI. In addition, we compare relative strains between DCM and healthy controls using 3-dimensional (3D) tagging, relating tissue motion characteristics to the temporal evolution of microstructure. Using biomechanical modeling, mechanistic insights into the underlying processes pertaining to the differences in fiber reorientation during cardiac contraction in patients with DCM versus controls are suggested.

## Methods

### Study Protocol

Patients with nonischemic DCM were enrolled at St. Thomas’ Hospital, King’s College London. Criteria for DCM were left ventricular ejection fraction (LVEF) <50%, no myocardial scar on the previous cardiac MR scan, and a previous invasive coronary angiogram confirming unobstructed epicardial coronary arteries. All patients were taking maximally tolerated medical therapy at the time of enrollment. Age-matched healthy volunteers without a history of cardiac events were enrolled as the control group at the University Hospital Zurich. Imaging was performed on 1.5T Philips Achieva systems (Philips Healthcare, Best, The Netherlands) equipped with 32-channel cardiac receiver arrays at both sites. Written informed consent was obtained from all subjects before imaging, and the study protocol was approved by the ethics committees of King’s College London and the Canton of Zurich. Obtained informed consent included imaging and publication of anonymized data.

Before diffusion imaging, balanced steady state–free precession cine data (spatial resolution 2×2×15 mm^3^ and temporal resolution 10 ms) were acquired in 2-chamber and short-axis view of the left ventricle (LV). On cine images, subject-specific mid-diastolic and peak systolic time points were visually determined. To assess cardiac function, a contiguous stack of short-axis cine images from apex to base was acquired (spatial resolutions 1.6×1.6×8 mm^3^ and temporal resolution 30 ms).

### Diffusion Tensor Imaging

Diffusion-weighted imaging was performed in short-axis orientation using stimulated echo acquisition mode imaging with single shot echo planar image readout.^23^ The imaging plane was placed at midventricular level, and the acquisition was ECG triggered to peak systole and mid-diastole. Consistent levels of breath holding were ensured by respiratory navigator gating (gating window 5 mm). Eight signal averages for each diffusion direction were acquired within a single breath hold. A total of 10 optimized diffusion directions were encoded^25^ with a *b* value of 350 s/mm^2^, resulting in 22 breath holds for 2 cardiac phases. Parameters of the diffusion sequence were as follows: field of view, 309×129 mm^2^; in-plane resolution, 2.5×2.5 mm^2^; slice thickness, 8 mm; Echo Time/Repetition Time, 18 ms/2 R–R intervals; and partial Fourier factor, 0.65.

### Motion Imaging

Tissue motion and strain were quantified using 3D complementary spatial modulation of magnetization tagged imaging, using a segmented echo planar imaging readout.^26^ Three orthogonally oriented line tagged cine image volumes were acquired sequentially, covering the whole LV. Data acquisition was navigator gated (acceptance window 15 mm) within 3 consecutive breath holds, each spanning over 18 heartbeats. Imaging parameters were as follows: field of view, 108×108×108 mm^3^; spatial resolution, 3.5×7.7×7.7 mm^3^; tag line distance, 7 mm (echo planar image factor 7, 3 excitations per heart phase); and temporal resolution, 20 ms. Geometric stack alignment of all tagged volume images was performed by incorporating navigator offsets and rigid image registration.

## Data Analysis

### Functional Analysis

Left and right ventricular volumes and ejection fractions and left ventricular mass were calculated by manually drawing end-diastolic and end-systolic contours on short-axis images, excluding the papillary muscles (CVI software, Circle Cardiovascular Imaging Inc, Alberta, Canada). Left ventricular wall thickness was measured at end diastole and end systole in the mid-LV, defined at the level of the papillary muscles.

### Diffusion Tensor Analysis

During image registration,^27^ systolic and diastolic diffusion tensors were determined taking diffusion weighting of the “*b*=0 s/mm^2^” image into account. Systolic diffusion tensors were corrected for myocardial strain as previously reported.^21^ During tensor calculation, HA, transverse, and sheet angles were computed as described in the Data Supplement.

For each diffusion tensor, a locally normalized transmural position was calculated. HAs were binned along 10 equidistant transmural positions, followed by linear regression to determine the slope of the transmural HA course. To avoid partial voluming effects at the endocardial and epicardial boundaries, data points from 80% of the inner myocardium were used for data fitting. HA ranges were computed as differences between maximum (endocardial) and minimum (epicardial) HA values within the 80% transmural interval. MD, FA, HA slopes, and transverse angles were evaluated in the whole LV for each cardiac phase and reported as median and interquartile ranges across both groups. Radial and axial diffusivities were computed similarly and are reported in the Data Supplement. Sheet angles were evaluated in the anterior septal region in the proximity of the surface coils to reduce the impact of noise as a confounding factor. Sheet angles are reported as histograms for both cohorts.

### Motion and Strain Analysis

On the 3D tagging data, endocardial and epicardial contours of the LV were manually defined while excluding papillary muscles. Longitudinal and circumferential strains were determined by contour tracking using the SinMod algorithm (TagTrack, GyroTools LLC, Zurich, Switzerland).^28^ Radial strain was evaluated by the Harmonic Phase algorithm.^29^ Cardiac torsion was normalized to long-axis length as described previously.^30^ Circumferential and radial strains are given in the midventricular region. Longitudinal strain was computed across the whole LV.

### Biomechanical Modeling

**Figure 1.**
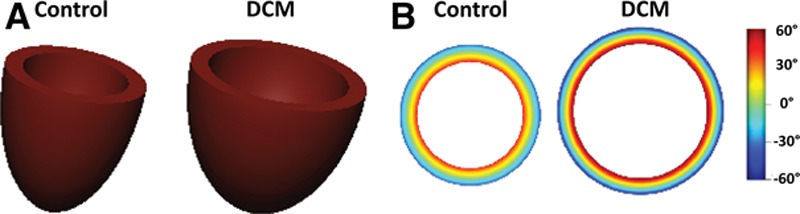
**A**, Idealized left ventricular models used for biomechanical modeling of healthy and dilated cardiomyopathy (DCM) hearts. **B**, Idealized helix angle maps based on average end-diastolic measurements in controls and patients with DCM.

Two idealized geometric models of the LV truncated at the base were created using the average measurements (end-diastolic long- and short-axis lengths, wall thicknesses, and cavity volumes). The 2 models represent an average of the control and DCM populations. Fiber distributions were chosen to match the average end-diastolic HA slopes observed in the respective cohorts. Figure 1 shows both models and the HA maps in short-axis view. Sheet angle (E2A) distributions were chosen to match the frequencies observed in the data at end diastole, with idealized transmural variation based on the previous studies^6,31^ changing from 0° to 90° between endocardium and midwall and from −90° to 0° between midwall and epicardium. Passive inflation and active contraction of the ventricle were simulated using the computational biomechanical models.^32–34^ The impact of dilatation and increased sphericity on the changes of HA slopes was explored and compared between the groups. In addition, strain and torsion were measured in both models with varying HA slopes to investigate whether the changes in fiber orientation were, in part, responsible for the differences seen in these parameters.

### Statistical Analysis

Differences between diastolic/systolic parameters and patients with DCM/controls were determined by a Wilcoxon signed-rank and Wilcoxon rank-sum test, respectively. A *P* value <0.05 was considered statistically significant.

## Results

In total, 15 patients and 10 healthy subjects were recruited. Nine patients and 9 healthy volunteers were scanned successfully and comprised the final cohort. Data sets were rejected because of either technical issues or breathing motion–related signal loss in the diffusion-weighted images. Demographics and clinical characteristics are given in Table 1. The DTI data are summarized in Table 2.

**Table 1.**
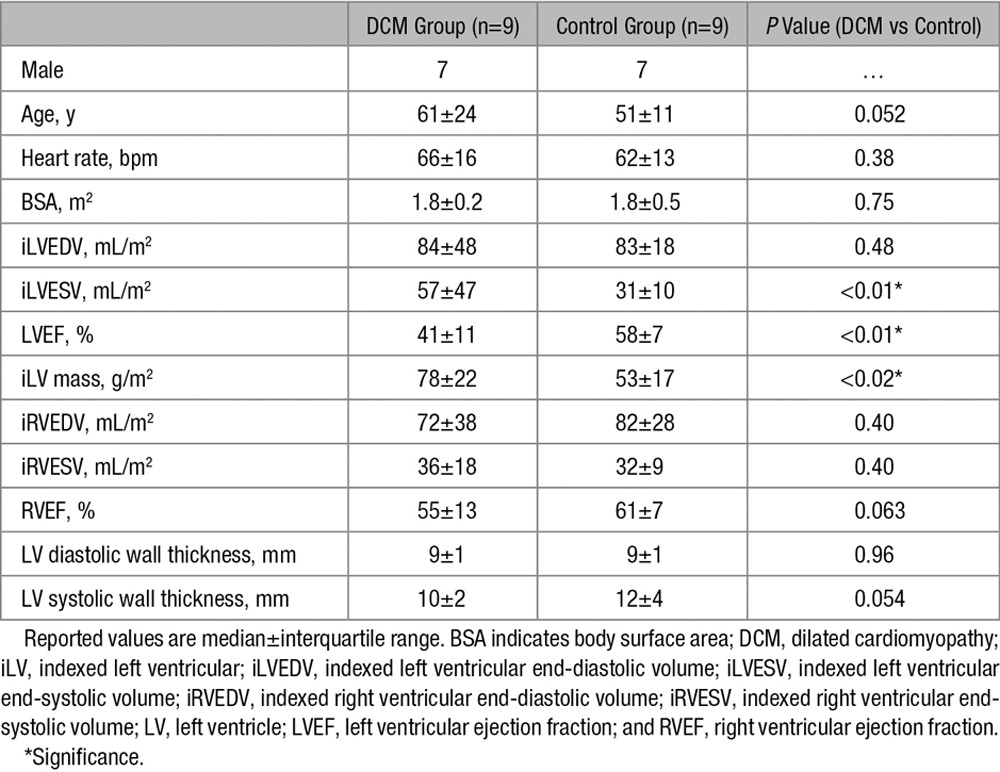
Patient Demographics and Data

**Table 2.**
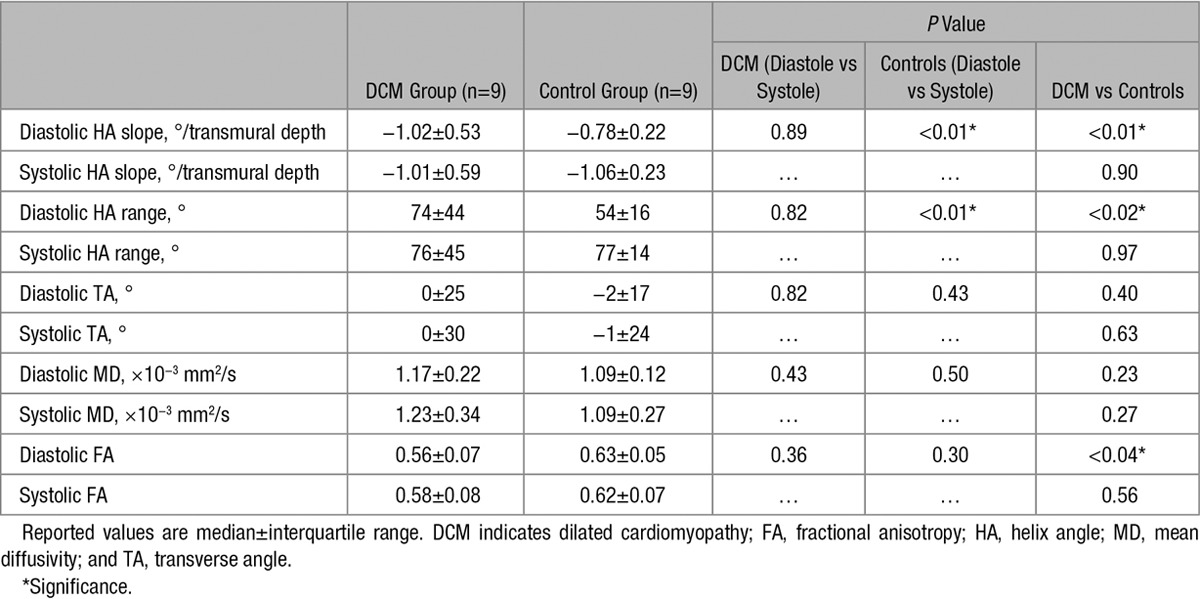
Diffusion Tensor Data

### Helix Angles

**Figure 2.**
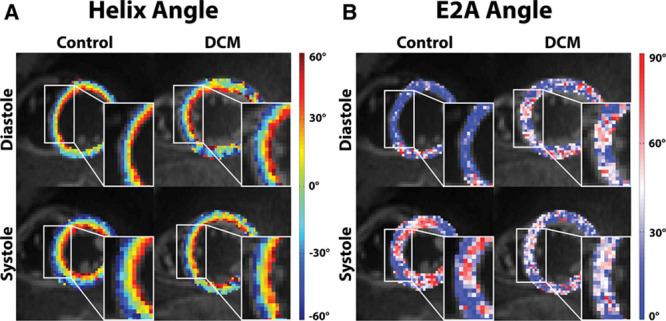
Comparison of helix angle (**A**) and E2A angle (**B**) maps acquired in diastole and systole from control vs patient with dilated cardiomyopathy (DCM).

**Figure 3.**
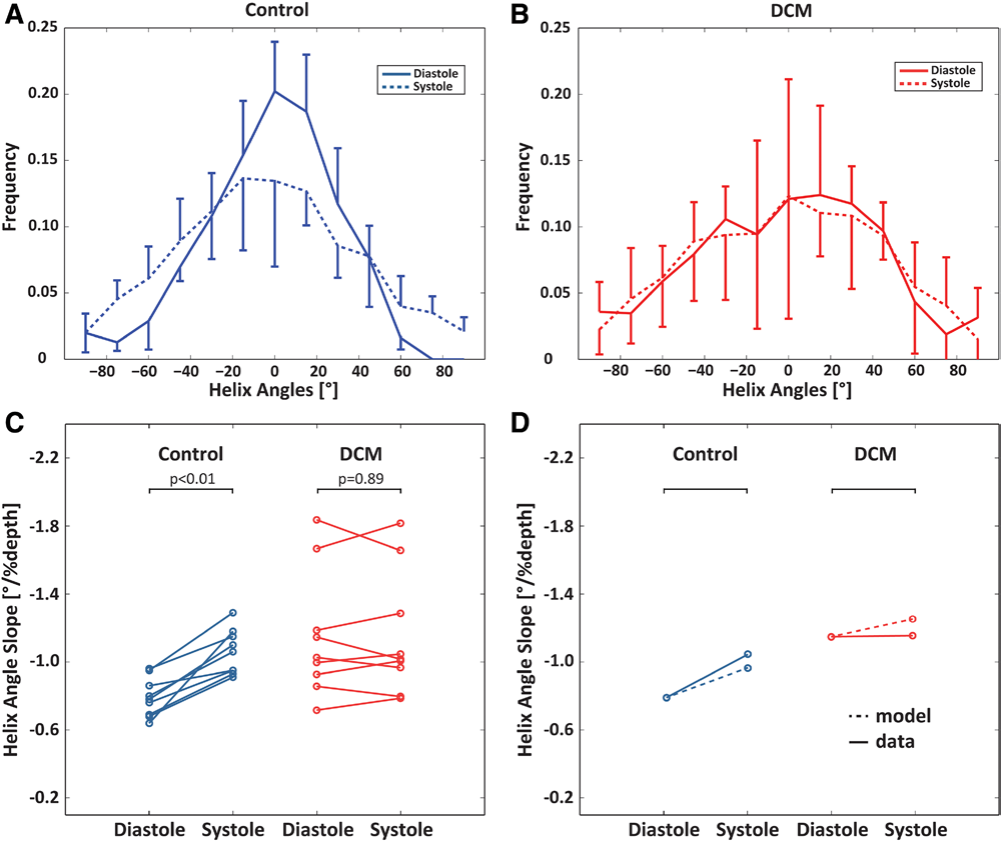
Histograms of diastolic and systolic helix angles for controls (**A**) and patients with dilated cardiomyopathy (DCM; **B**). Although a shift toward steeper helix angles is seen in the systolic healthy heart, systolic and diastolic distributions are similar in the DCM case. Error bars indicate interquartile ranges across the subjects. **C**, Corresponding transmural helix angle slopes in diastole vs systole for the control and DCM groups. **D**, Diastolic and systolic helix angles for control and DCM modeling when compared with the data.

Figure 2 shows an example of HA and sheet (E2A) angle maps for a control subject and patient with DCM in diastole and systole. Histograms of HA distributions for both cohorts are displayed in Figure 3A and 3B. A change toward steeper HAs during contraction is seen in the controls, whereas inconsistent dynamic change between heart phases is observed in the patients. Bin counts of diastolic HAs close to 0° are reduced in patients with DCM relative to healthy controls. The corresponding transmural HA slopes are shown in Figure 3C.

In diastole, the HA slope was significantly steeper in the patients when compared with controls (−1.02±0.53°/% depth versus –0.78±0.22°/% depth; *P*<0.01).

In systole, there was a significant increase in maximum endocardial and epicardial HA in the controls, indicating a more longitudinal alignment of myofiber aggregates with cardiac contraction. This resulted in a significant increase in HA slope in the control group from diastole to systole (−0.78±0.22°/% depth to −1.06±0.23°/% depth; *P*<0.01). In contrast, there was no significant change in HA slope from diastole to systole in the patients with DCM (−1.02±0.53°/% depth to −1.01±0.59°/% depth; *P*=0.89).

### Transverse Angles

Transverse angles are distributed around 0°, indicating the expected circumferential alignment of the fiber aggregates (Table 2).

### Sheet Angles

**Figure 4.**
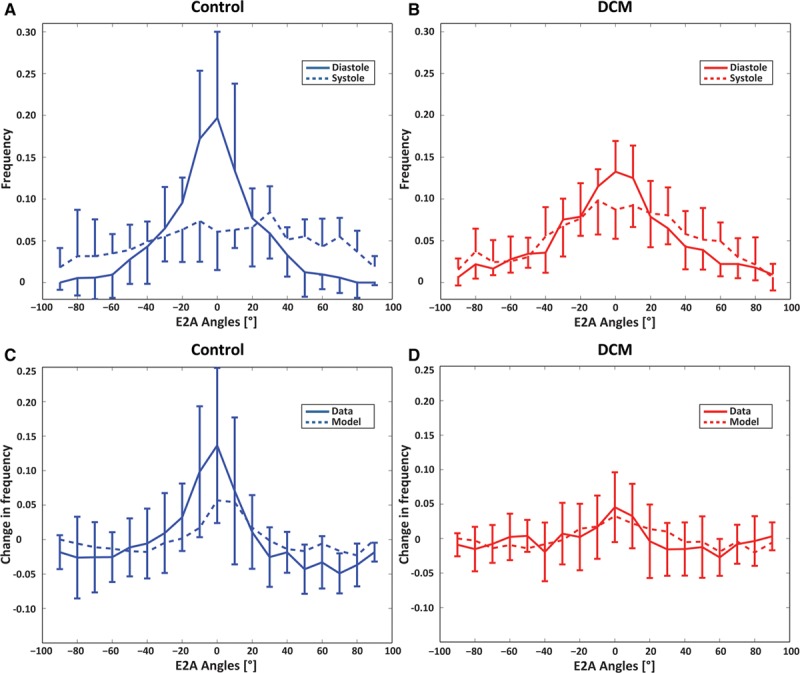
Histograms of diastolic and systolic E2A sheet angles for controls (**A**) and patients with dilated cardiomyopathy (DCM; **B**). The sheet angle distribution is broader in the systolic healthy heart compared with diastole, whereas systolic and diastolic distributions are similar in the DCM case. Histograms of change between diastolic and systolic E2A distributions for controls (**C**) and patients with DCM (**D**). Controls exhibit a marked change in E2A as opposed to little change in patients with DCM. Model results follow a similar trend. Error bars indicate interquartile ranges across the subjects.

Histograms of E2A sheet angle distributions for controls and patients with DCM are shown in Figure 4A and 4B. A change in E2A angles toward a broader distribution during contraction is seen in the control group, whereas reduced dynamic change between diastole and systole is observed in the patients with DCM. Bin counts of diastolic sheet angles close to 0° are reduced in DCM relative to healthy controls.

### Mean Diffusivity and Fractional Anisotropy

In both diastole and systole, there was lower FA in the DCM group than in the controls (diastole 0.56±0.07 versus 0.63±0.05, respectively; *P*<0.04 and systole 0.58±0.08 versus 0.62±0.07, respectively; *P*=0.56). There were no significant differences in FA between cardiac phases in either group.

There was a trend toward higher MD in the DCM group relative to controls (diastole 1.17±0.22×10^−^^3^ mm^2^/s versus 1.09±0.12×10^−^^3^ mm^2^/s; *P*=0.23 and systole 1.23±0.34×10^−^^3^ mm^2^/s versus 1.09±0.27×10^−^^3^ mm^2^/s; *P*=0.27). There were no significant differences in MD between cardiac phases in either group.

### Torsion and Strain

**Figure 5.**
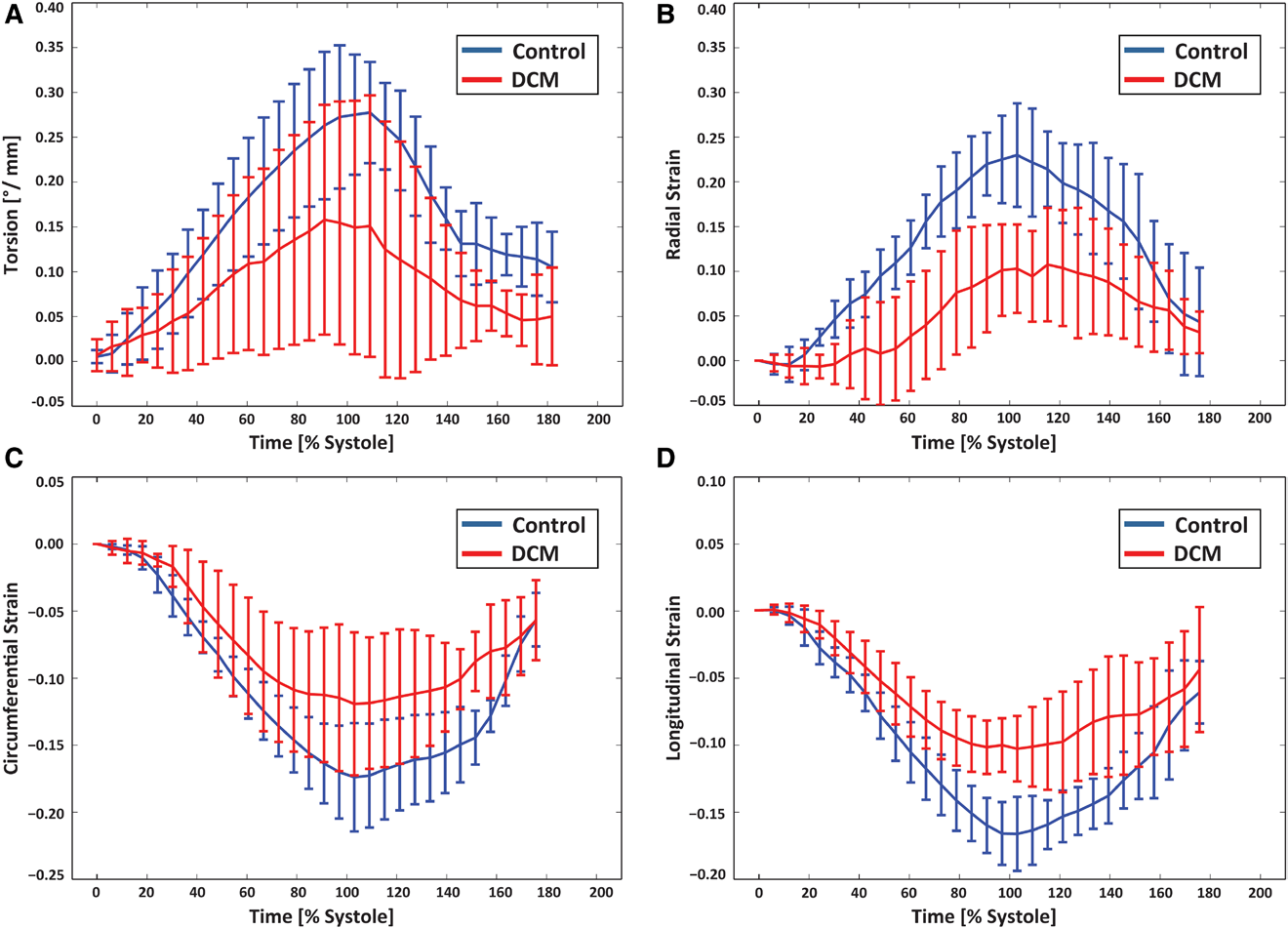
Time course of myocardial torsion (**A**), radial (**B**), circumferential (**C**), and longitudinal (**D**) strain for dilated cardiomyopathy (DCM) and control. Error bars indicate interquartile ranges across the subjects.

Table 3 reports maximum torsion and strain for both groups. Figure 5 shows the median torsional deformation and cardiac strain parameters during the cardiac cycle. Longitudinal, circumferential, and radial strains were all significantly reduced in the DCM group compared with the controls.

**Table 3.**
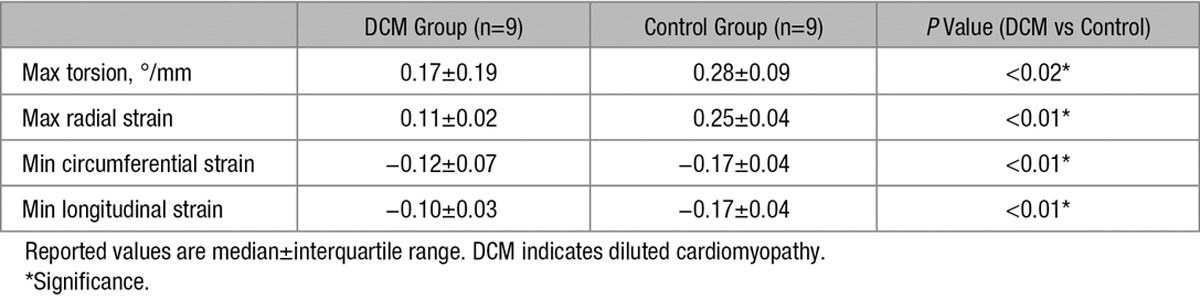
Torsion and Strain Data

**Figure 6.**
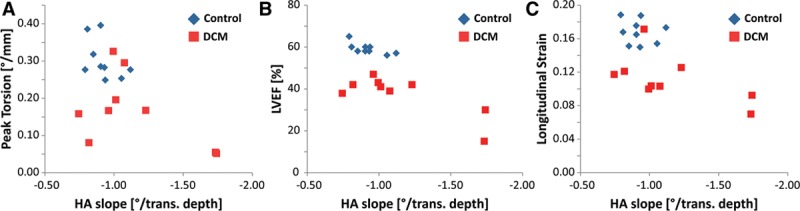
Peak torsion (**A**), left ventricular ejection fraction (LVEF; **B**), and (negative) longitudinal strain (**C**) as a function of normalized helix angle (HA) slope. A trend toward lower torsion (**A**), LVEF (**B**), and longitudinal strain (**C**) with increasing HA slope is seen in the dilated cardiomyopathy (DCM) group.

Mean (systolic and diastolic) HA slope was correlated against maximum torsion, LVEF, and longitudinal strain. The correlations were found to be limited in both the groups (Figure 6). In the patients, there was a trend toward reduced torsion and longitudinal strain with increased HA slope compared with controls. LVEF remained on constant level of around 40% for HA slopes of ≈ -1.0/% transmural depth, however decreased to 15% with steepened HA slope.

In the control group, values for peak torsion were spread by ≈15% around 0.30°/mm, and the values for longitudinal strain were in the range of 0.15 to 0.19 for the controls compared with 0.07 to 0.17 for the patients with DCM. LVEF values in controls were densely distributed around 58±7%.

### Biomechanical Modeling

The active contraction phase was simulated for the healthy and the DCM models to understand the differences in the HA changes between end-diastolic and end-systolic states. Both models were progressively activated at respective end-systolic volumes, and the change in HA slopes at the endocardial and epicardial surfaces was recorded. Figure 3D shows the results of the 2 models. The observation from the data that in controls, the slope change is higher than in patients with DCM is supported by the model. The change in E2A distributions between diastole and systole is illustrated in Figure 4C and 4D. A significant change is observed in controls (≤0.13/0.06 in data/model), whereas the distribution for patients with DCM remains largely unchanged (≤0.03/0.03 in data/model), reflecting the trend seen in the data.

**Figure 7.**
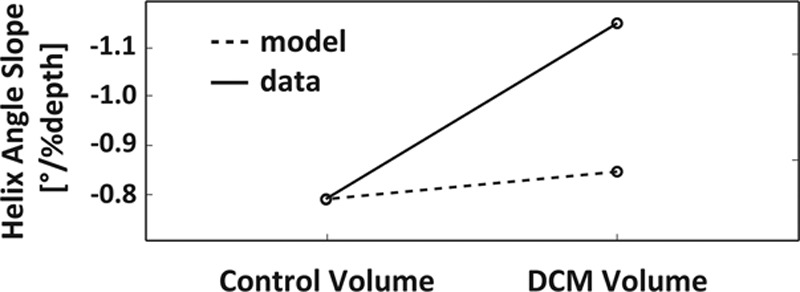
Change in helix angle slope caused by passive inflation of the control left ventricular model to dilated cardiomyopathy (DCM) model cavity volume when compared with measured difference in control and DCM helix angle slopes. Volume change alone does not explain observed differences between the 2 cohorts.

The next modeling objective was concerned with potential explanation of the observed changes in HAs by the dilatation and remodeling seen in DCM hearts. To this end, the control LV was inflated from its reference volume to the DCM model reference volume. The measured HAs at the larger volume are steeper, but the change is too low to explain the difference between the measured control and DCM HA slopes (0.03°/%depth; Figure 7). When repeated for the DCM LV, a more spherical geometry, and inflated to twice the cavity volume, a larger change in HA was observed (0.1°/%depth). However, the DCM modeled result was still significantly lower than the values observed in the data (0.36°/%depth difference calculated between control and DCM DTI data). This suggests that LV size and shape do not seem to have a significant impact on the steepening of HAs seen in patients with DCM compared with controls.

Finally, we aimed to investigate whether the change in HA was contributing to the reduced torsion and strain or was compensatory in preserving cardiac function. Strain and torsion were evaluated in each model with both control and DCM HA distributions. The results are shown in Table 4. The results show that in the DCM group, when simulating with steeper angles, torsion was reduced and strain values were unchanged. A similar pattern was demonstrated in the control group. This suggests that steeper angles do not play a compensatory role in aiding cardiac contraction, and that torsion impairment is exacerbated with steeper angles in hearts of either geometry. It is worth noting that the values of strain and torsion do not directly compare to those from the data, although there are clearly similar trends and this likely reflects the use of an idealized nonpatient-specific model.

**Table 4.**
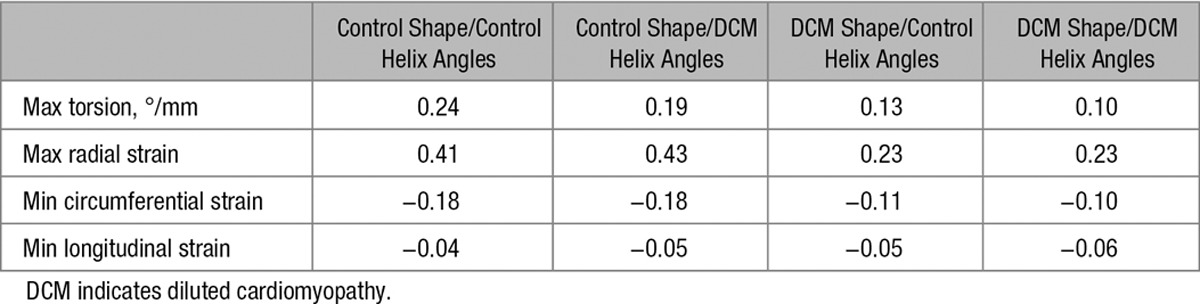
Biomechanical Modeling

## Discussion

In this study, the dynamic change of myofiber aggregate orientation in patients with DCM, and healthy controls was investigated using in vivo dual-phase cardiac DTI. A statistically significant change of myocyte orientation between diastole and systole was found in the control group. The longitudinal fiber alignment during contraction is assumed to optimize cardiac pumping efficiency and has been described previously using in vivo and ex vivo DTI data and histology.^21,24,35–37^ In contrast to healthy controls, the change in HA from diastole to systole was found to be far less pronounced in patients with DCM and was not statistically different between both heart phases. On average, diastolic myofiber aggregate orientation had a more longitudinal orientation in patients with DCM relative to healthy controls.

Seven of 9 patients with DCM showed comparable systolic HA configurations relative to healthy controls. In the 2 remaining patients, however, clearly, increased HA slopes in both cardiac phases were measured. It is speculated that this elevated longitudinal myocyte orientation could be the result of advanced remodeling because these 2 patients were considerably older (76 and 77 years) when compared with the mean age of the patient population (61±24 years) and had the poorest LVEF. In accordance, successive elongation of myocytes and reduced contractility may have been present. Similar reorientation was previously described by Tseng et al^13^ in hypertrophic hearts, however not confirmed by more recent data.^10^

In addition, we demonstrate that there are differences in sheet angle pattern between the groups. In the controls, sheet angle distribution changes dynamically between systole and diastole with a broader distribution in systole. However, the systolic and diastolic distributions in the patients with DCM are similar. A comparable pattern has been demonstrated in patients with hypertrophic cardiomyopathy.^10^

In general, cardiac pumping performance in the patients with DCM was significantly reduced compared with the controls as quantified by several parameters: LVEF, maximum torsion, longitudinal, radial and circumferential strain. In particular, low LVEF, torsion and strain values were found for the 2 patients with elevated HA slopes. Our data on maximum torsion in patients with DCM and controls are in good agreement with the literature values.^38^

As well as the clinical and gross structural changes seen in DCM, the results of myofiber aggregate reorientation must be considered alongside well-recognized subcellular changes, such as microarchitecture disarray. Histologically, heart failure is characterized by myocyte elongation, reduced myocyte density, and fibrosis, and these changes have been correlated with ex vivo DTI results in the previous studies.^18,39^ The observed trend toward increased MD and decreased FA supports the presence of reduced myofibril density and cellular necrosis. Myocardial perfusion can contribute to additional signal attenuation in diffusion-weighted images and may bias diffusion metrics. On the basis of the values derived by Scott et al,^40^ the impact of perfusion on MD was simulated. With a *b* value of 40 s/mm^2^ for the reference image (“*b*0”), MD is overestimated by ≈11%; however, this value has only been established in the context of healthy subjects, and further study would be needed in a DCM cohort.

Using the biomechanical modeling, we were able to reproduce similar trends between the model and the data. There were differences of changes in HA when activating the model relative to the in vivo findings prompting for further work, including patient-specific geometries and myofiber aggregate data and myocyte modeling at the microscale. The use of biomechanical modeling was 2-fold: first, to try to understand whether the size and shape of the ventricle could be responsible for the altered steepness of the myofiber aggregates in diastole. Second, implications of the fiber alterations were investigated, in particular, whether they are beneficial or counterproductive to efficient cardiac contraction. Initial tests showed that even pronounced dilatation of the heart is not sufficient to produce the observed differences in HA between the groups. We, however, noted that the agreement of simulated and actually measured myofiber aggregate orientation improves, when simulating a dilated, more spherically shaped heart. Furthermore, the biomechanical simulations show that the reduction in torsion seems to be exacerbated by steeper HAs with no improvement or deterioration in strain. This was found in both LV models used, suggesting that this factor is independent of the changes to LV size and shape. In accordance, even in a dilated and remodeled heart, the steeper angles are not beneficial to maintaining longitudinal, radial, or circumferential strains or torsion. The underlying reasons for exacerbated deterioration in strain and torsion observed in the DCM group may, thus, reflect underlying subcellular changes that could not be examined in this study.

There is little correlation with any of the single parameters tested against the change in HA slope in the subjects studied. This suggests that a composite of factors, such as dilatation, poor systolic function, and the recognized other subcellular alterations seen in DCM, may be responsible for this structural rearrangement. Further studies would be needed to confirm this and may be a future area of study.

In summary, we have been able to demonstrate that there are changes in diastolic myocyte orientations and, importantly, that there is reduced and inconsistent dynamic reorientation during cardiac contraction in patients with DCM relative to healthy hearts. We have been able to show that the change in left ventricular shape does not entirely explain the difference seen in HA slope between patients with DCM and controls. We have also demonstrated findings that suggest that steeper HAs do not confer a compensatory adaptation in terms of cardiac contraction. Overall, our findings provide new insights into the structural alterations within the living heart in DCM and underline the importance of MR DTI to gain deeper understanding of cardiac disease.

### Study Limitations

The DCM patient cohort in this study had a diagnosis of nonischemic cardiomyopathy; however, the cause may be heterogeneous. Clinical, morphological, and functional data were variable, and, accordingly, a large variation of HA distribution was seen. Overall, the patients studied present a form of milder DCM, and, therefore, our results cannot provide a complete picture of changes in myofiber aggregate architecture in all stages of DCM. Similarly, the impact of medical therapy cannot be commented on because of the small sample size, but this would be an interesting avenue of future work. Furthermore, some of the measurements were taken only in specific regions of the myocardium to reduce low signal-to-noise effects and, therefore, may not be representative of the entire myocardium should there be heterogeneous changes.

Because of scan time constraints, the imaging resolution of cardiac DTI was relatively coarse (2.5×2.5×8 mm^3^), and hence partial voluming effects were inherently present at the endocardial and epicardial borders. To reduce the impact of this, edge voxels were excluded from the analysis. In line with the limited scan time available, only 1 slice could be acquired using cardiac DTI.

## Sources of Funding

We acknowledge funding from UK Engineering and Physical Sciences Research Council (EP/I018700/1 and EP/N011554/1), the Swiss National Science Foundation grant number 320030_153014, Adult Congenital Heart Disease Service Guy’s and St. Thomas’ and the National Institute for Health Research Biomedical Research Centres at Guy’s and St. Thomas’ National Health Service Foundation Trust, King’s College London and University College London Hospitals. Dr Lamata holds a Sir Henry Dale Fellowship jointly funded by the Wellcome Trust and the Royal Society (grant number 099973/Z/12/Z).

## Disclosures

None.

## Supplementary Material

## CLINICAL PERSPECTIVE

Dilated cardiomyopathy (DCM) is a multifactorial disease, heterogeneous in cause, but histologically most findings are nonspecific. Despite significant developments, DCM is not yet fully characterized and prognosis remains poor. This is the first study demonstrating the feasibility of in vivo cardiac magnetic resonance diffusion tensor imaging with myocardial tagging in patients with DCM to explore the dynamic reorientation of myofiber aggregates coupled with the contractile properties of the heart. We demonstrate a more longitudinal orientation of diastolic myofibers in patients compared with controls. In contrast to controls, consistent myocyte reorientation during systole was absent in patients. These findings are associated with a worsening in strain and torsion, providing a connection of microstructural and macrostructural alterations seen in DCM. Biomechanical modeling of the data suggests that though left ventricular remodeling seems to be an important factor in the changes to myocyte orientation, other mechanisms are also at play. The altered arrangement of myofiber aggregates does not appear to be compensatory for the reduction in contractility in DCM. This is clinically relevant because many changes in heart failure, such as fluid retention and sympathetic overdrive, are known to be maladaptive responses. The changes seen in diffusivity and diffusion anisotropy are suggestive of findings, such as cellular necrosis. DCM is known to be a slowly progressing condition. Interestingly, we show that older patients with lower ejection fraction had steeper helix angles and lower longitudinal strain and torsion. This preliminary finding supports further work, suggesting that diffusion tensor imaging may carry potential as a biomarker to define disease severity.
