# Supplementary material for: Studying Dynamic Myofiber Aggregate Reorientation in Dilated Cardiomyopathy Using In Vivo Magnetic Resonance Diffusion Tensor Imaging
Source: Circ Cardiovasc Imaging. 2016 Oct 18;9(10):e005018. doi: 10.1161/CIRCIMAGING.116.005018 (PMC5068188; doi:10.1161/CIRCIMAGING.116.005018)
Supplement: Supplementary file 1 [file hci-9-e005018-s001.pdf]

Supplemental Material A

The helix angle α reflects the local helix elevation, i.e. the angle between the projection of the first eigenvector of the diffusion tensor onto the epicardial surface and the transmural plane. The transverse angle β denotes the deviation of the helix from the circumferential orientation and, accordingly, is defined as the angle between the projection of the first eigenvector onto the short-axis plane and the circumferential contour (Figure S1a).

The E2A sheet angles were computed according to Ferreira et al. [1]. To this end, the cross-myocyte plane perpendicular to the projection of the first eigenvector onto the epicardial surface ($E1_{proj}$) was determined for each voxel. Subsequently, the second eigenvector (E2) was projected onto this plane and the angle relative to the cross-myocyte direction (orthogonal to radial direction and $E1_{proj}$) was measured.

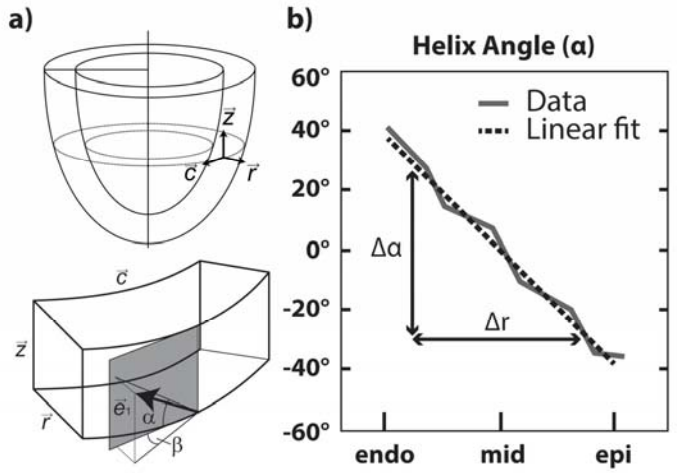

Figure S1: a) Defintion of helix angle (α): The angle between the projection of the first eigenvector onto the epicardial surface and the transmural plane. b) Corresponding linear fit of the transmural course of the helix angles.

Supplemental Material B

Overview of the biomechanical model

The biomechanical model is based on the classical continuum approach combined with finite element discretisation. This supplement provides a brief overview of the mathematical and computational model. Further particulars of the model and methods used can be found in [2,3].

Let the reference configuration of the myocardium be denoted by Ω_0 with the coordinate $\boldsymbol{X}$, and a deformed configuration at time $t \in (0,T]$, $T>0$, be denoted by Ω_t with the coordinate $\boldsymbol{x}$. At a given time t domain deformations can be defined as $\boldsymbol{u} = \boldsymbol{x} - \boldsymbol{X}$, while the hydrostatic pressure and the boundary tractions (described below) are denoted by p and $\boldsymbol{\lambda}$ respectively. The principle of stationary potential energy [4] states that at any time t this deformation can be found by minimising the total energy of the system:

$$\Pi(\boldsymbol{u},p,\boldsymbol{\lambda}) = inf_{v} sup_{q,\mu} \Pi(\boldsymbol{v},q,\boldsymbol{\mu}).$$

The total energy can be separated into internal and external energy terms:

$$\Pi = \Pi_{int}(\boldsymbol{u},p) + \Pi_{ext}(\boldsymbol{u},p,\boldsymbol{\lambda}).$$

We assume that myocardium can be modelled as a hyperelastic incompressible tissue, so that the internal energy can be written in terms of the strain energy function Ψ, the hydrostatic pressure p and the determinant of the deformation gradient $J = det(\boldsymbol{F})$, where $\boldsymbol{F} = \nabla_{\boldsymbol{X}}\boldsymbol{u} + \boldsymbol{I}$, and $\boldsymbol{I}$ is the identity matrix:

$$\Pi_{int} = \int_{\Omega 0} \Psi + p(J - 1)\, d\boldsymbol{X}.$$

The strain energy function incorporates orthotropic passive and active behaviour of the tissue:

$$\Psi = \Psi_p + \Psi_a.$$

The model employs the Holzapfel-Ogden passive constitutive law [5], defined as follows:

$$\Psi_p = a / (2b) \exp[b(I_1 - 3)] + \sum_{i=f,s} a_i \;/ (2b_i) (\exp[b_i(I_{4i} - 1)^2] - 1) + a_{fs} / (2b_{fs}) \exp[b_{fs} I_{8fs}^2],$$

where $a, b, a_f, b_f, a_s, b_s, a_{fs}, b_{fs}$ represent material parameters (values given in Table S2 below), and $I_1, I_{4f}, I_{4s}, I_{8fs}$ are the strain invariants associated with fibre and sheet directions. Specifically, if at a given location in the reference domain the myocyte aggregate (referred to as fibre in the model) and sheet orientation vectors are $\boldsymbol{f_0}$ and $\boldsymbol{s_0}$ respectively, and $\boldsymbol{C} = \boldsymbol{F}^T \boldsymbol{F}$ is the right Cauchy-Green strain tensor, then

$$I_1 = \boldsymbol{C} : \boldsymbol{I}, \quad I_{4f} = \boldsymbol{f_0} \cdot (\boldsymbol{C}\, \boldsymbol{f_0}), \quad I_{4s} = \boldsymbol{s_0} \cdot (\boldsymbol{C}\, \boldsymbol{s_0}), \quad I_{8fs} = \boldsymbol{f_0} \cdot (\boldsymbol{C}\, \boldsymbol{s_0}).$$

Active response is produced with a simplified version of Kerchoffs-type length-dependent constitutive laws [6,7] with reference myocyte compressive strain l_0=0.8, global active tension value *AT*, and added transverse activation at 30% of the fibre activation as discussed in [8,9]:

$$\Psi_a = AT \int_0^{I4f} \tanh(2(\sqrt{\xi}-l_0))\, d\xi + 0.3\, AT \int_0^{(I1-I4f)} \tanh(2(\sqrt{\xi}-l_0))\, d\xi.$$

The external energy Π_{ext} comes from any forces acting on the boundaries of the domain. Ventricular cavity volume is set via an endocardial energy term

$$\Pi_{endo} = \lambda_{endo}(V-V_{data}),$$

where λ_{endo} is a scalar endocardial Lagrange multiplier representing cavity pressure, V the cavity volume produced by the simulation, and V_{data} the prescribed cavity volume. The cavity volume can be approximated as follows [2]:

$$V = - \int_{\Gamma endo} 0.5\, [(\boldsymbol{I} - \boldsymbol{n}_b \otimes \boldsymbol{n}_b)\, \boldsymbol{x}] \cdot \boldsymbol{n}\, d\boldsymbol{x},$$

where Γ_{endo} denotes the deformed configuration of the endocardial surface of the ventricle, $\boldsymbol{n}_b$ is the base normal vector and $\boldsymbol{n}$ the outward endocardial normal vector.

A simpified base condition allowing sliding in plane only was imposed due to the generic nature of the model. The base plane was aligned with the z = 0 plane, and the centre of the base coincided with the origin. The external energy term on the base could be written as follows:

$$\Pi_{base} = \int_{\Gamma base} \lambda_{base} (\boldsymbol{u} \cdot \boldsymbol{n}_b)\, d\boldsymbol{x} \;+\; \lambda_{0,1} \int_{\Gamma base} u_1\, d\boldsymbol{x} + \lambda_{0,2} \int_{\Gamma base} u_2\, d\boldsymbol{x} + \lambda_2 \int_{\Gamma base} u_1 \cdot x_2\, d\boldsymbol{x}\,,$$

where Γ_{base} denotes the deformed configuration of the base surface of the ventricle, λ_{base} is a spatially varying Lagrange multiplier enforcing no longitudinal motion of the base plane, $\boldsymbol{n}_b$ is the base normal vector, $\lambda_{0,1}$ and $\lambda_{0,2}$ are scalars ensuring no translation of the base centre, and λ_2 is a scalar enforcing no rotation around the axis.

With this definition of the energy terms, and combining all Lagrange multipliers in a vector $\boldsymbol{\lambda}=(\lambda_{endo},\lambda_{base},\lambda_{0,1},\lambda_{0,2},\lambda_2)$, the full state of the system $(\boldsymbol{u},p,\boldsymbol{\lambda})$ at a given time t is found as the critical point of the total energy Π:

$$D_{(\boldsymbol{u},p,\boldsymbol{\lambda})}\, \Pi(\boldsymbol{u},p,\boldsymbol{\lambda}) = 0.$$

This equation provides the weak form of the problem, which can be solved numerically using the finite element method. The reference domain is discretised into quadratic hexahedral elements, with Q2-Q1 approximation for the displacement-pressure pair, quadratic approximation on quadrilateral surface elements for λ_{base}, and constant approximations for λ_{endo}, $\lambda_{0,1}$, $\lambda_{0,2}$ and λ_2. The resulting nonlinear system is solved via Newton-Raphson iteration with line search, with linear solve steps carried out by direct matrix inversion.

All simulation results were obtained using *C*Heart [10], a parallel multiphysics software engine.

Test specifications

Two reference geometries were produced: one to represent a generic healthy ventricle, and another to represent a generic DCM ventricle. Both shapes were simplified as ellipsoids cropped in the short axis plane below the base of the ventricle. The shapes were adjusted in such a way that passive inflation to prescribed end-diastolic volume produced representative short and long axis dimensions and wall thickness. The prescribed cavity volume, both at end diastole and end systole, was set to values lower than the data averages to account for ventricle truncation. The end-systolic state for each geometry was obtained by prescribing end-systolic volume, and gradually increasing active tension in the tissue to reach end-systolic cavity pressures of ~100 mmHg. It should be noted that the ventricle dimensions, as well as the HA/E2A values undergo significant changes at the first few activation steps (AT < 25 kPa), and then stabilise, meaning that the precise cut-off pressure (with AT > 100 kPa) has virtually no effect on these measurements. The prescribed and observed metrics are presented in Table S1.

	Control			DCM		
	Reference state (REF)	End diastole (ED)	End systole (ES)	Reference state (REF)	End diastole (ED)	End systole (ES)
Cavity volume, ml	50	92	37	82	162	100
LA, cm	6.40	7.74	7.35	6.40	7.58	7.15
SA, cm	4.20	5.13	3.53	5.40	6.83	5.74
WT, mm	12.0	8.9	11.6	12.0	8.5	10.0

Table S1. Dimensions of the idealised geometrical models used in simulations (LA/SA – long/short axis length, WT – wall thickness). Low cavity volumes are the result of ventricle truncation. Cells with grey/white background show metrics that were prescribed/obtained using simulations.

Parameters used in passive inflation (given in Table S2) were based on values obtained for a healthy volunteer in [11]. Further, the values of the stiffness parameters a, a_f, a_s, a_{fs} were scaled to produce realistic end-diastolic pressures (10-15 mmHg) and ventricle dimensions similar to those observed in the data.

	a, kPa	b	a_f, kPa	b_f	a_s, kPa	b_s	a_{fs}, kPa	b_{fs}
Control	0.24	3.0	4.80	4.0	0.96	1.5	0.4	3.4
DCM	0.60	3.0	6.40	4.0	1.28	1.5	0.6	3.4

Table S2. Parameters of the Holzapfel-Ogden constitutive law used in the simulations.

Supplemental Material C

Mean Diffusivity (MD) and Fractional Anisotropy (FA) Maps

Upon tensor reconstruction, MD and FA maps were computed for DCM and control (Figure S2).

MD and FA are defined as follows:

$$MD = \frac{\lambda_1 + \lambda_2 + \lambda_3}{3} \quad \text{and} \quad FA = \sqrt{\frac{3}{2}} \frac{\sqrt{\sum_{i=1}^{3} (\lambda_i - MD)^2}}{\sqrt{\sum_{i=1}^{3} \lambda_i^{\,2}}}$$

With the three eigenvalues of the diffusion tensor: $\lambda_1, \lambda_2, \lambda_3$.

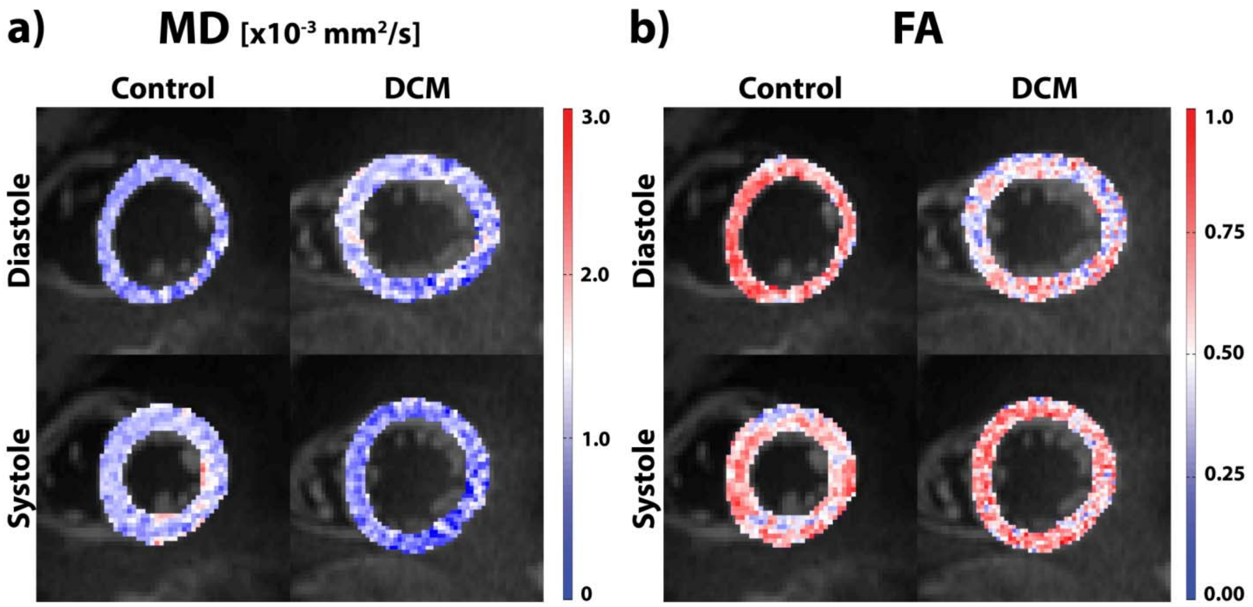

Figure S2: Example MD (a) and FA (b) maps for DCM and control.

Representative Raw Data

Figure S3 shows example raw data images for DTI (b=0, b=400 s/mm^2) and 3D tagging data.

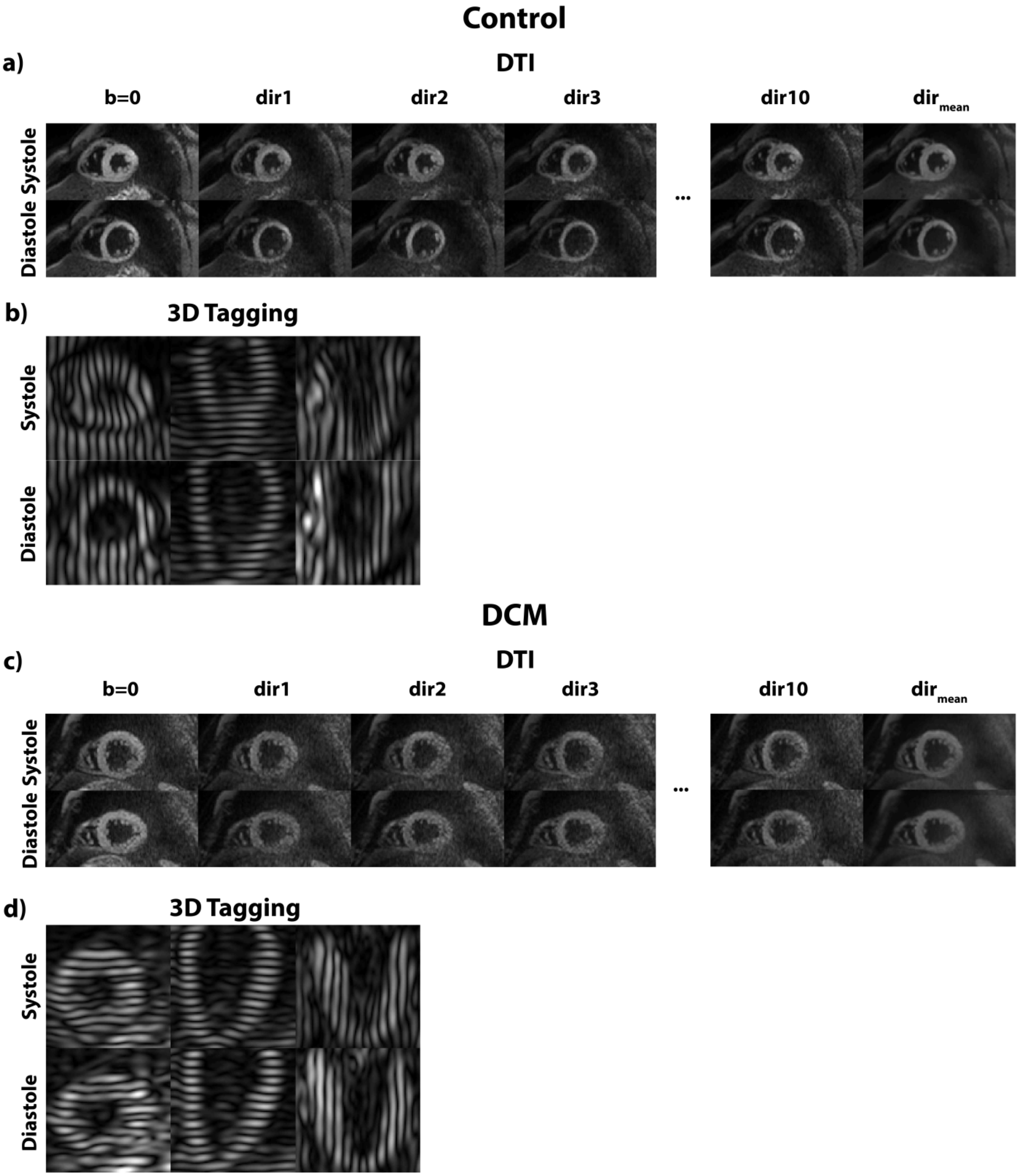

Figure S3: Example raw data images for Control (a,b) and DCM (c,d).

Supplemental Material D

Axial ($\lambda_{\parallel}$) and radial ($\lambda_{\perp}$) diffusivities were computed in the left ventricle for both cohorts and heart phases. They are defined as follows:

$$\lambda_{\parallel} = \lambda_1 \text{ and } \lambda_{\perp} = \frac{\lambda_2 + \lambda_3}{2}$$

With the three eigenvalues of the diffusion tensor: $\lambda_1, \lambda_2, \lambda_3$. Table S3 reports $\lambda_{\parallel}$ and $\lambda_{\perp}$ as median and interquartile ranges across both groups. DCM diffusivities were found to be increased or at least equal to corresponding diffusion metrics of the control group. Diastolic diffusion data is in very good agreement with previous data [12], while systolic data shows an increase in axial and radial diffusivity for DCM compared to controls. Differences in diffusivities between DCM and control were determined by Wilcoxon rank sum testing.

According to Tsagalou et al [13], DCM patients suffer from depressed coronary flow reserve and reduced capillary density compared to healthy subjects. Hence the impact of perfusion on radial and axial diffusivities is expected to be reduced in DCM relative to controls. It is speculated that actual diffusivities (without perfusion bias) for the control group may result in clearer statistical significances between both cohorts.

Diffusivity [x10^{-3}mm^2/s]	Diastole		Systole	
	Axial ($\lambda_{\parallel}$)	Radial ($\lambda_{\perp}$)	Axial ($\lambda_{\parallel}$)	Radial ($\lambda_{\perp}$)
Control	1.9±0.2	0.6±0.1	1.9±0.3	0.7±0.3
DCM	1.9±0.3	0.8±0.3	2.1±0.4	0.8±0.2
p (Control vs. DCM)	0.86	0.03	0.23	0.31

Table S3. Axial and radial diffusivities for control and DCM.

References

1. Ferreira P, Kilner PJ, McGill L-A, Nielles-Vallespin S, Scott AD, Spottiswoode BS, Zhong X, Ho SY, McCarthy K, Ismail T, Gatehouse P, Silva R, Lyon A, Prasad SK, Firmin D, Pennell DJ. In vivo cardiovascular magnetic resonance diffusion tensor imaging shows evidence of abnormal myocardial laminar orientations and mobility in hypertrophic cardiomyopathy. J. Cardiovasc. Magn. Reson. 2014;16:P338.
2. Asner L, Hadjicharalambous M, Chabiniok R, Peressutti D, Sammut E, Wong J, Carr-White G, Chowienczyk P, Lee J, King A, Smith N, Razavi R, Nordsletten D. Estimation of passive and active properties in the human heart using 3D tagged MRI. Biomech. Model. Mechanobiol. 2016;15:1121-39.
3. Asner L, Hadjicharalambous M, Chabiniok R, Peressutti D, Sammut E, Wong J, Carr-White G, Lee J, King A, Smith N, Razavi R, Nordsletten D. Patient-specific modeling for left-ventricular mechanics using data-driven boundary energies. Comput. Method Appl. M. Epub August 11, 2016. DOI http://dx.doi.org/10.1016/j.cma.2016.08.002
4. Bonet J, Wood R. Nonlinear continuum mechanics for finite element analysis. Cambridge University Press, Cambridge. 2008.
5. Holzapfel G A, Ogden R W. Constitutive modelling of passive myocardium: a structurally based framework for material characterization. Philos. Trans. Ser. A. Math. Phys. Eng. Sci. 2009; 367:3445–3475.
6. Kerckhoffs R C P, Bovendeerd P, Prinzen F, Smits K, Arts T. Intra-and interventricular asynchrony of electromechanics in the ventricularly paced heart. J. Eng. Math. 2003; 47:201–216.
7. Niederer S A, Plank G, Chinchapatnam P, Ginks M, Lamata P, Rhode K S, Rinaldi C A, Razavi R, Smith N P. Length-dependent tension in the failing heart and the efficacy of cardiac resynchronization therapy. Cardiovasc. Res. 2011; 89:336–343.
8. Usyk T P, Mazhari R, Mcculloch A D. Effect of laminar orthotropic myofiber architecture on regional stress and strain in the canine left ventricle. J. Elast. 2001; 61:143–164
9. Usyk T P, Legrice I J, Mcculloch A D. Computational model of three-dimensional cardiac electromechanics. Comput. Vis. Sci. 2002; 4:249–257.
10. Lee J, Cookson A, Roy I, Kerfoot E, Asner L, Vigueras G, Sochi T, Deparis S, Michler C, Smith N P, Nordsletten D A. Multiphysics Computational Modeling in CHeart. SIAM J. Sci. Comput. 2016; 38:C150-C178.
11. Gao H, Carrick D, Berry C, Luo X Y. Parameter estimation of the Holzapfel–Ogden law for healthy myocardium. J. Eng. Math. 2015; 1–18.
12. Abdullah OM, Drakos SG, Diakos N, Wever-Pinzon O, Kfoury AG, Stehlik J, Selzman CH, Reid BB, Brunisholz K, Verma DR, Myrick C, Sachse FB, Li DY, Hsu EW. Characterization of diffuse fibrosis in the failing human heart via diffusion tensor imaging and quantitative histological validation. NMR Biomed. 2014;27:1378–86.
13. Tsagalou EP, Anastasiou-Nana M, Agapitos E, Gika A, Drakos SG, Terrovitis JV, Ntalianis A, Nanas JN. Depressed Coronary Flow Reserve Is Associated With Decreased Myocardial Capillary Density in Patients With Heart Failure Due to Idiopathic Dilated Cardiomyopathy, JACC 2008;52:1391-8
